# Fetal Atrial Septal Aneurysm: Follow-Up from Second to Third Trimester

**DOI:** 10.3390/diagnostics12061469

**Published:** 2022-06-15

**Authors:** Roxana Gireadă, Alexandra Ursache, Roxana Matasariu, Răzvan Socolov

**Affiliations:** Department of Obstetrics and Gynecology, University of Medicine and Pharmacy “Gr. T. Popa”, 700115 Iaşi, Romania; rox.gireada@gmail.com (R.G.); socolov.razvan@gmail.com (R.S.)

**Keywords:** atrial septal aneurysm, prenatal screening, fetal echocardiography

## Abstract

Atrial septal aneurysm (ASA) is a rarely reported fetal finding. Its definition is variable, but the diagnosis is usually made when the foramen ovale flap extends at least halfway across the left atrium. It is considered a transient, self-limiting condition, but on occasion, it can be complicated by fetal arrhythmia or left ventricular (LV) inflow obstruction—if longstanding, this can lead to left heart hypoplasia. We present two cases of ASA diagnosed at the second trimester scan, one of which was subsequently complicated by LV inflow obstruction and prenatal suspicion of hypoplastic aortic arch. This report is a good illustration of how structure follows function: a small LV preload can lead to a decreased LV output, which in turn will end up in a hypoplastic LV and outflow tract—all this is reversible after birth, due to the physiological circulatory modifications that occur in the newborn.

**Figure 1:**
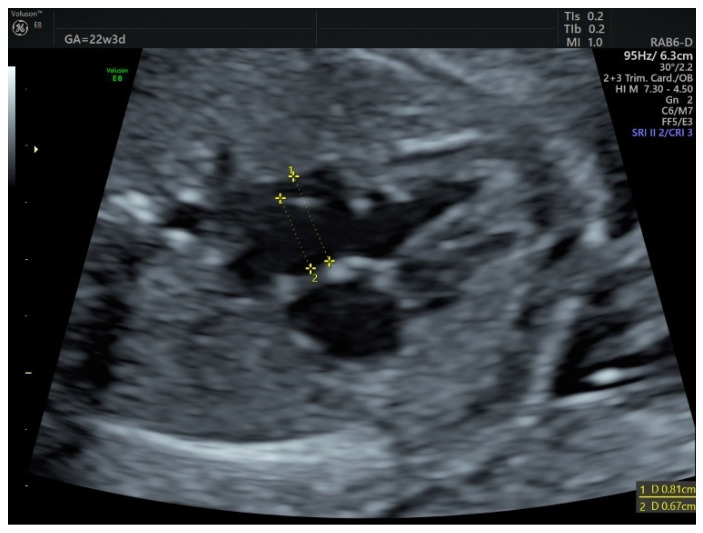
Atrial septal aneurysm (ASA), also known as foramen ovale aneurysm, aneurysm of septum primum, and redundant septum primum flap, is usually diagnosed when the foramen ovale flap is hypermobile, extending at least halfway across the left atrium, in a balloon appearance. To measure this hypermobility, the atrial septal excursion index (ASE index) can be calculated as the ratio between the maximum displacement of the atrial septum and the left atrium transverse diameter [1]. The exact prevalence of fetal ASA is not known. Most cases go unreported since this finding is isolated and the evolution uneventful. A more accurate depiction of its prevalence can be extrapolated from newborn series: from 1072 consecutive echocardiograms performed in the early postnatal period, the prevalence of ASA was 7.6% and went up to 11.1% in preterm newborns [2]. Fetal ASA is considered a benign finding, just a transitory phase in the natural history of foramen ovale closure [3,4]. Even though it has a high-resolution rate, ASA should be followed-up prenatally due to its possible complications: fetal cardiac arrhythmias and left ventricle (LV) inflow obstruction. Coarctation of the aorta was also sometimes observed alongside ASA, but there is no known association between these two conditions [1]. ASA-associated arrhythmias consist mostly of premature atrial contractions (PAC), which can sometimes progress to supraventricular tachycardia [5,6,7]. If the foramen ovale flap is very redundant, it can make a cyclical contact with the mitral valve and even protrude in the LV, thus obstructing its inflow. This obstruction can progress to LV hypoplasia and aortic arch hypoplasia [8,9,10]. However, the outcome is usually favorable even in such cases, due to the hemodynamic changes brought about by the first breaths of the newborn. The postnatal normalization of cardiac structures could be explained by the increased pulmonary venous return, which in turn increases the left atrium filling and normalizes the atrial septal position [8], thus correcting the LV preload and output and eventually leading to a normal filling of the aorta. The possibility of changing fetal cardiac physiology was also demonstrated in a small series of ASA cases associated with left heart hypoplasia, where short-term maternal hyperoxygenation induced immediate changes in LV geometry and promoted an anterograde flow through the aortic arch [1]. After birth, ASA is associated with a higher risk of incomplete foramen ovale closure [2], so postnatal echocardiography is formally recommended. Although it is not uncommon to find an ASA in the third trimester, especially with advancing gestation [11], to our knowledge, it has never been reported in the second trimester. We present two cases of ASA diagnosed in the second trimester, and their follow-up in the third trimester. The progression to left heart hypoplasia in one of our cases is a good illustration of how structure fits function—a small LV preload can lead to a decreased LV output, which in turn will end up in a hypoplastic LV and hypoplastic LV outflow tract, but physiological functional changes in the newborn circulation can restore normal cardiac structure. Therefore, during prenatal counseling of suspected LV/aortic arch hypoplasia, one must keep in mind obstructive ASA as a differential diagnosis since the outcome is almost always spontaneously favorable after birth. The first case is of a 33-year-old G4P1, without priors, that presented at 22w3d for her second trimester screening scan. The ultrasound showed a balloon appearance of the foramen ovale flap (ASE index = 0.82), with normal LV inflow. The atrial septal aneurysm was followed up in the third trimester, without notable complications (no PACS, no LV hypoplasia). A healthy baby girl weighing 3750 g was delivered vaginally at 39w. Six months after birth, incomplete closure of the foramen ovale was demonstrated on echocardiography, in the form of a 2 mm interatrial communication. ASE index, atrial septal excursion index; LV, left ventricle; PAC, premature atrial contraction.

**Figure 2:**
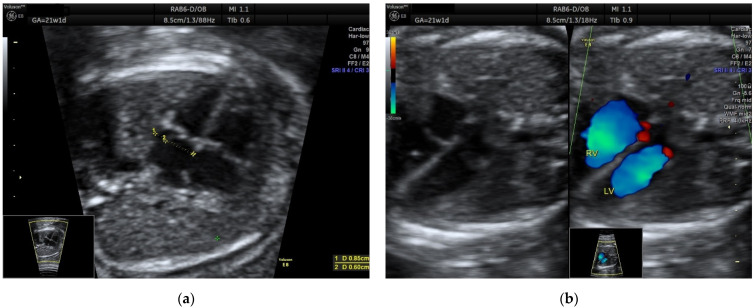

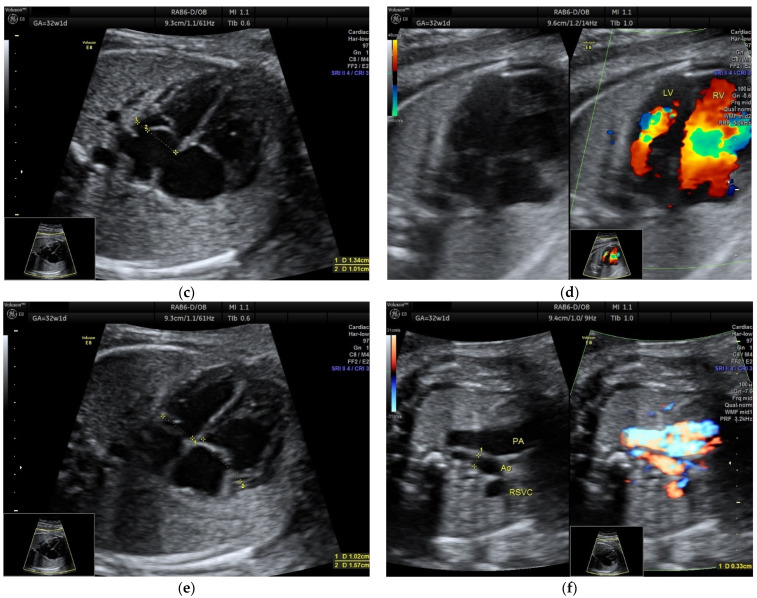
The second case is a 24-year-old G1P0, without priors, that presented at 21w1d for her second trimester screening scan. An atrial septal aneurysm was also demonstrated (ASE index = 0.70) (**a**), with normal LV inflow (**b**). At 32w1d, the foramen ovale flap was making a cyclical contact with the mitral valve, almost covering it (ASE index = 0.75) (**c**), and significantly reducing the LV inflow (**d**). This diminished preload had led to a thinner LV (cardiac chamber asymmetry, RV/LV = 1.53) (**e**). The aortic arch had an anterograde flow but was significantly smaller than in the second trimester (aortic isthmus Z-score = −2.03) (**f**), supporting further evidence of reduced LV output (Video S1). No PACs were noted until delivery. Due to the suspected aortic arch hypoplasia, the patient delivered in a third-level maternity, where neonatal cardiovascular surgery was available on site. A baby boy weighing 3690 g was delivered by C-section at 39 w, with good postnatal adaptation. The transthoracic echocardiography performed in the early neonatal period showed normally sized LV and aortic arch, while the foramen ovale closed incompletely, with a double interatrial communication. There was also a suspicion of aortic valve malformation, which was not confirmed at the 6 months follow-up, when only a 2.5 mm patent foramen ovale was present. ASE index, atrial septal excursion index; LV, left ventricle; RV, right ventricle; PAC, premature atrial contraction; PA, pulmonary artery; Ao, aorta; RSVC, right superior vena cava.

## Data Availability

Not applicable.

## Supplementary Materials

The following supporting information can be downloaded at: https://www.mdpi.com/article/10.3390/diagnostics12061469/s1, Video S1: Obstructive fetal atrial septal aneurysm.
